# Interplays of Interfacial Forces Modulate Structure and Function of Soft and Biological Matters in Aquatic Environments

**DOI:** 10.3389/fchem.2020.00165

**Published:** 2020-03-17

**Authors:** Motomu Tanaka

**Affiliations:** ^1^Physical Chemistry of Biosystems, Institute of Physical Chemistry, Heidelberg University, Heidelberg, Germany; ^2^Center for Integrative Medicine and Physics, Institute for Advanced Study, Kyoto University, Kyoto, Japan

**Keywords:** soft matter, biosystems, interfacial forces, disjoining pressure, specular reflectivity

## Abstract

Water had been considered as a passive matrix that merely fills up the space, supporting the diffusion of solute molecules. In the past several decades, a number of studies have demonstrated that water play vital roles in regulating structural orders of biological systems over several orders of magnitude. Water molecules take versatile structures, many of which are transient. Water molecules act as hydrogen bond donors as well as acceptors and biochemical reactions utilize water molecules as nucleophiles. Needless to say, the same principle holds for the synthetic materials that function under water: the conformation, dynamics and functions of molecules are significantly influenced by the surrounding water. This review sheds light on how the structure and function of soft and biological matter in aquatic environments are modulated by the orchestration of various interfacial forces.

## Introduction

Water shares about 60–65 wt% of an adult human body, whose most prominent example is cytosolic fluid inside ~100 trillion (10^14^) cells making up our body. Mounting evidence suggests that water is not a simple continuum supporting passive diffusion of solute molecules. On the contrary, water plays more active roles in controlling the conformation and dynamics of biopolymers and proteins over several orders of magnitude both in space and time (Ball, 2017). For example, ^2^H spin relaxation studies on bacteria cultured in D_2_O showed that about 85 % of water in bacteria has bulk-like dynamics (τ ~ 10^−11^ s), and the dynamics of the rest of water is slower by one order of magnitude (Persson and Halle, 2008). Most strikingly, a very small fraction of water (~0.1 %) shows a significantly slow dynamics with τ ~ 10^−6^ s. Thus, if one considers the interafacial interactions between water and biological matter, the interactions inevitably involve both free (bulk) and bound (hydrating) water.

**Figure 1:**
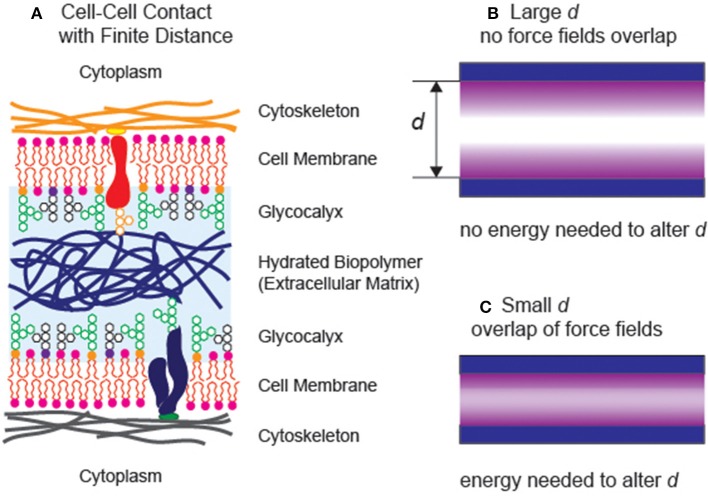
**(A)** Schematic illustration of two neighboring cells sustaining a finite distance. **(B)** A simple model of two surfaces separated at a large separaction distance *d*. Both interfaces can be treated as independent (free) interfaces, as the force fields do not reach each other. **(C)** The additional energy is necessary to move the distance between two planes when the force fields overlap with each other.

If we look into biological systems, a variety of interfacial interactions are combined to sustain living systems in water (Alberts, 2017). For example, epithelial cells establish stable, specific contacts with neighboring cells. On the other hand, cells in connective tissues, both in loose and dense connective tissues, hardly make any contacts with their neighbors. The intercellular space is filled with various biopolymers, acting as “cushions” and “lubricants” to help tissues withstand compressional and frictional stresses, respectively (Figure 1A). Moreover, the layer of oligo- and polysaccharides coating the outer surface of cell membranes, called glycocalyx, plays major roles in avoiding non-specific binding of cells. The glycocalyx of vascular endothelial cells does not only act as a lubricating layer reducing the hydrodynamic frictions to the blood flow but also avoid non-specific adhesion (clotting) of erythrocytes, leukocytes, and platelets. Since the structure and function of biomolecules near the interface are distinctly different from those in bulk aqueous solution, the fine-adjustment of the balance between strong, specific interactions and generic, weak interactions is the key to optimize the biointerface and maximize the functions of biomolecules.

In chemistry and material sciences, the basic understanding on interactions of soft matter with interfacial is highly relevant, too. For example, the surfaces of contact lenses and inplants must remain inert by avoiding non-specific deposition (fouling) of proteins and cells (Hucknall et al., 2009). On the other hand, polymeric substrates used for the cell culture need to be adhesive, in order that the cells and adhere and proliferate. More recently, ample evidence suggests that the elasticity of hydrogel substrates significantly influence the shape, motion, and function of biological cells (Discher et al., 2005), implying that the density of crosslinks and hence the degree of hydration are key factors to regulate cellular functions.

The main focus of this review is to provide the readership with an overview on how the interplay of various interfacial forces physically regulates the strucctures and functions of soft and biological matters operating in aquatic environments. Here, the term “aquatic” is used to describe the systems whose physical properties are modulated by water near the interface, which is different from the molecules dissolved in “aqueous” solutions in bulk.

## Basic Concept: Disjoining Pressure

In general, if the interlayer between two planes takes a finite distance, the interlayer still possesses its intrinsic bulk properties (Figure 1B), two interfaces can be described independently within the framework of the Gibbs capillary theory. When the phase volume and interface area are constant, a change in the separation distance does not alter the total free energy of the system. On the other hand, once the long-range force fields in the thin interlayer overlap (Figure 1C), any change in the separation distance would cost certain energy penalties. This work, which can be positive or negative, primarily originates from the long-range, interfacial forces including (i) van der Waals forces, (ii) hydration repulsions, (iii) electrostatic forces, and (iv) entropic (steric) forces. Albeit each of the contributing forces has been studied in simpler model systems, the quantitative determination of the interplay of individual forces in complex, biological systems.

About one century ago, Derjaguin introduced the concept of “disjoining pressure” that is a difference between the pressure (force acting on a unit area) of a phase in bulk and the pressure of the same phase in the vicinity of the surface (Derjaguin and Churaev, 1987). From the thermodynamic viewpoint, this is nothing but the first derivative of the Gibbs free energy with respect to the distance in the direction perpendicular to the surface:

$$\begin{array}{r} \Pi \left(d\right)=-{\left(\frac{\partial G}{\partial d}\;\right)}_{T,\mu }. \end{array}$$

Note that ∂*G* is nothing but the work required to alter the distance between two planes by ∂*d*. Namely, under thermodynamic equilibrium, the finite distance between two planes, corresponding to the free energy minimum can be found at ${\left(\frac{\partial^{2}G}{\partial d^{2}}\right)}_{T,\mu }>0$ and hence ${\left(\frac{\partial \Pi }{\partial d}\right)}_{T,\mu }<0$. On the other hand, the interface becomes unstable when ${\left(\frac{\partial \Pi }{\partial d}\right)}_{T,\mu }>0$, leading to a continuous thinning of the interlayer that finally results in the dissipation.

## Interaction With Water: Hydration

One characteristic behavior of biopolymers in aquatic environments is “hydration (swelling).” The pressure generated by hydration, called hydration repulsion, originates from the work required to remove water from a hydrated layer to the bulk liquid phase. This can be given as an exponential decay function over distance,

$$\begin{array}{r} \Pi_{hyd}=\Pi_{hyd}^{0}exp\left(-\frac{d}{\lambda }\;\right). \end{array}$$

In general, the disjoining pressure Π(*d*) can be determined experimentally by measuring the equilibrium layer thickness (*d*) under external pressure. For example, the characteristic decay length λ can be calculated by measuring the layer thickness at different osmotic pressures, $\Pi_{osm}=\frac{kT}{V_{H2o}}\ln\;h_{rel}$, by using various experimental techniques. *V*_H2O_ is the molar volume of water and *h*_rel_ the relative humidity.

**Figure 2:**
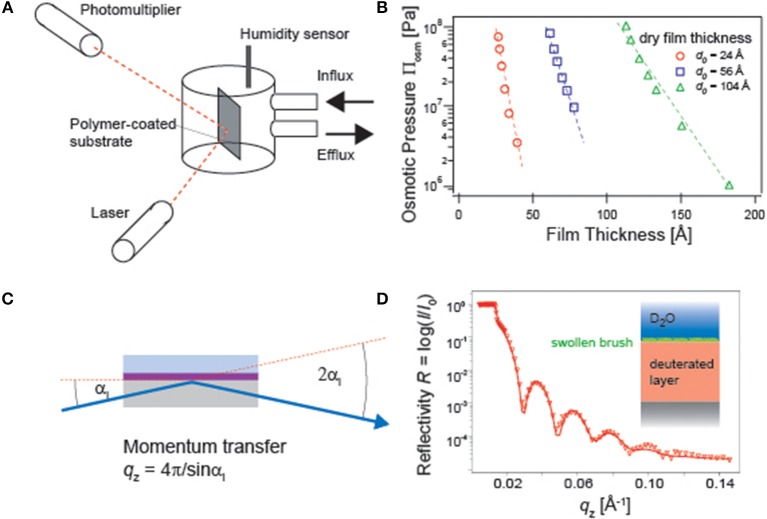
**(A)** Ellipsometry experiments under controlled humidity conditions. The relative humidity and hence the osmotic pressure inside the measurement chamber can be controlled by mixing dry air and air saturated with water vapor. **(B)** The obtained pressure-thickness can be used to obtain $\Pi_{hyd}^{0}$ and the characteristic decay length λ (Rehfeldt and Tanaka, 2003). **(C)** Specular neutron reflectivity setup for a polymer film in water. **(D)** A reflectivity curve of polymer brushes. Use of D_2_O and a deuterated polymer substrate enhances the scattering length density contrast (Rehfeldt et al., 2006).

For example, the influence of osmotic pressure on the distance between phospholipid membranes can be quantified by small-angle X-ray scattering measurements under various osmotic pressures (Leneveu et al., 1976; Rand and Parsegian, 1989). Micro-interferometry and specular X-ray reflectivity have also been used to monitor how the atmospheric osmotic pressure influences the thickness of free-standing films of surfactants and block copolymers (Guenoun et al., 1995; Bergeron et al., 1996). Ellipsometry coupled to a humidity chamber (Figure 2A) can also be used to measure the change in polymer film thickness and refractive index of polymer thin films deposited on solid substrates, yielding the pressure-distance relationship (Rehfeldt and Tanaka, 2003; Wong et al., 2004) (Figure 2B). However, once polymer chains are immersed in bulk water, the osmotic pressure equals to zero. Naturally, the polymer chains are more swollen and possess larger conformational degrees of freedom. A higher degree of hydration and hence a higher volume fraction of water ϕ_water_ shifts the total refractive index from that of dry polymer *n*_dry_ to that of water *n*_water_, because *n*_*swollen*_ = *n*_*dry*_(1 − ϕ_*water*_) + *n*_*water*_ϕ_*water*_. This makes the use of ellipsometry practically difficult.

One of the commonly used methods to probe the structures of hydrated polymer films perpendicular to the surface is specular X-ray and neutron reflectivity (Russell, 1990) (Figure 2C). The refractive index, *n* = 1 − δ + *i*β, contains the real and imaginary parts. As the latter corresponds to the absorption, β = 0 for a non-magnetic matter. The real part can be written as

$$\begin{array}{r} \delta =\frac{{\lambda^{2}N}_{A}\rho b_{mono}}{2\pi M_{mono}}, \end{array}$$

where λ stands for the wavelength of neutron beam, *N*_A_ the Avogadro's constant, ρ the density, *b*_mono_ the scattering length summed up for all atomic nuclei over one monomer, and *M*_mono_ the molecular weight of one monomer. Among the typical elements in polymers, the scattering length of proton (−3.74 × 10^−15^ m) is clearly different from that of deuteron (6.67 × 10^−15^ m). There, specular neutron reflectivity is highly suited for the structural investigation of materials swollen by water, as one can gain higher contrast by using either hydrogenated polymers in D_2_O or deuterated polymers in H_2_O (Figure 2D).

The intensity of the reflected beam was measured at each incidence angle α_i_ and hence momentum transfer in the direction perpendicular to the interface, $q_{z}=\frac{4\pi }{\lambda }\sin\alpha_{i}$. Note that the reflectivity must be corrected by the footprint of the beam as well as by the beam intensity at each incidence angle. The reflectivity of stratified films can be treated with the classical Abellé's matrix formalism and Parratt's recursive method (Abelès, 1950; Parratt, 1954). To deal with a realistic, not perfectly flat interface, a gradual transition of the density can be modeled using an error function with a root-mean-square (r.m.s.) roughness. After the correction, the amplitude of the reflection coefficient for each layer (slab) reads as

$$\begin{array}{r} r_{0,1}=\;r_{0,1}^{Fresnel}exp\left[-\frac{1}{2}q_{z}^{2}\sigma^{2}\;\right], \end{array}$$

where $r_{0,1}^{Fresnel}$ is the Fresnel reflection coefficient of an ideally flat interface (Névot and Croce, 1980).

However, such a “slab model” is no longer applicable to deal with highly swollen polymers, as the r.m.s. roughness becomes comparable to or larger than the layer thickness. The density profile of highly swollen polymer films in contact with water can be characterized by using a parabolic function (Milner et al., 1988; Kuhl et al., 1998) or an exponential function with decay length Λ and stretching factor *h* (Schneck et al., 2009; Rossetti et al., 2015),

$$\begin{array}{r} \rho \left(z\right)\propto exp{\left(-\frac{z}{\Lambda }\;\right)}^{2h}. \end{array}$$

Alternatively, the density profile function can be obtained analytically by splitting the density profile into thin slabs corresponding to the instrumental resolution (Pedersen and Hamley, 1994; Miller et al., 2005). It is notable that it is no longer necessary to include the roughness, since the slab thickness is so thin. Finally, the density of each slab was varied to achieve the minimum χ^2^ deviation between measured data and simulations.

## Polymer-Supported Membranes as the Model of Biointerfaces

**Figure 3:**
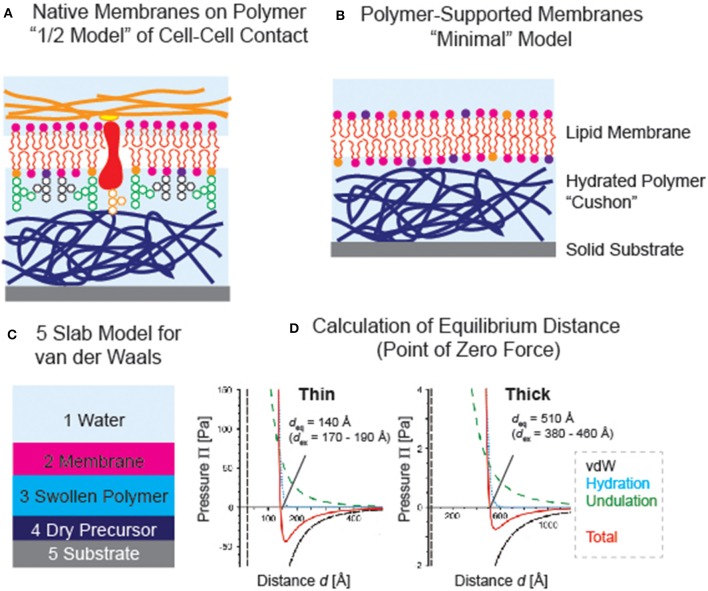
**(A)** Polymer-supported membranes as “1/2 model” of cell-cell contact (see Figure 1A). **(B)** “Minimal model” based on a phospholipid membrane deposited on a polymer support. **(C)** Example of van der Waals model; 5 slab model used for polymer-supported membranes. **(D)** Calculated van der Waals, hydration repulsion, and membrane undulation forces. Equilibrium membrane-substrate distance coincides with the point of zero force at which the sum of three forces (red) crosses Π = 0 (Rossetti et al., 2015). The results culculated for thin (dry thickness ≈ 40 Å) and thick (dry thickness ≈ 160 Å) films are presented, showing good agreements with the values determined by specular neutron and X-ray reflectivity experiments. Note that the counter balance between van der Waals attraction and hydration repulsion plays dominant roles in confining the membrane position.

For about 25 years now, hydrated polymer thin films have been utilized as the support for planar phospholipid membranes, which mimics the generic functions of extracellular matrix (Tanaka and Sackmann, 2005). They serve as “soft cushions” and “lubricative layers” and separate the membrane from the underlying solid substrates. This significantly reduces the risk of non-specific protein-substrate contacts that that might cause the denaturation of proteins. Although various polymer supports, such as chemically immobilized (or cross-linked) gels (Kühner et al., 1994; Kuhl et al., 1998), polymer brushes (Rehfeldt et al., 2006; Kaufmann et al., 2011), and polyelectrolyte multilayers (Wong et al., 1999; Delajon et al., 2005), have been reported, not all hydrophilic polymer films could act as good supports for membranes. Since it is even possible to spread “native cell membranes” uniformly on polymer cushions (Tanaka et al., 2004) or spread cell membrane extracts mixed with lipopolymer-containing vesicles (Pace et al., 2015), such systems can be considered as a “1/2 model” of cell-cell contact (Figure 3A). One of the most important criterion in choosing the polymer material is that the polymer-supported membranes must be thermodynamically stable. Actually, several reports have shown the instability or phase separation of membranes on polymer supports (Elender et al., 1996; Smith et al., 2009). This naturally calls for fine-adjustment of the interfacial interactions.

However, it is experimentally very difficult to quantify individual forces acting in complex, stratified systems under water. Therefore, it is better to start from a relatively simple case: a membrane of zwitterionic phospholipids on a uncharged polymer supports deposited on a silicon wafer (Figure 3B) (Rossetti et al., 2015). The electrostatic interaction between SiO_2_ and the membrane is screened by using a buffer close to the physiological condition (e.g., 10 mM phosphate buffer with 100 mM NaCl) and a 10–100 nm thick neutral polymer film, because the Debye screening length (<10 Å) is more than one order of magnitude smaller than the polymer thickness. In this case, we can avoid a relatively complicated consideration of electrostatic interactions at the polymer-membrane interface and focus on three forces, i.e., hydration repulsion (explained above), van der Waals, and entropic undulation force.

For hydration repulsion, $\Pi_{hyd}^{0}$ and λ can be determined experimentally by measuring the equilibrium thickness of the polymer films under different osmotic pressures using ellipsometry (Rehfeldt and Tanaka, 2003).

The van der Waals force can be calculated by using an asymmetric multilayer model (Israelachvili, 1992). For instance, the model for five layers (Figure 3C) should read:

$$\begin{array}{l} P_{vdW}\;=\frac{1}{6\pi }[\frac{A_{234}}{d^{4}}-\frac{\sqrt{A_{121}A_{343}}}{{\left(d_{2}+d_{3}\right)}^{3}}-\frac{\sqrt{A_{323}A_{545}}}{{\left(d_{3}+d_{4}\right)}^{3}}\\ \;+\frac{\sqrt{A_{545}A_{121}}}{{\left(d_{2}+d_{3}+d_{4}\right)}^{3}}]. \end{array}$$

*A*_ijk_ stands for the Hamaker constant representing interactions between layer *i* and *k* across layer *j, d*_2_ the thickness of the lipid membrane, *d*_3_ the thickness of the hydrated polymer and interfacial water, and *d*_4_ the sum of the thickness of hydrophobic precursor or poorly hydrated layer. The Hamaker constants were estimated by reviewing the data published in the literature for similar systems. The determination of the Hamaker constants for polymer- and membrane-containing interfaces require some assumptions, as discussed in previous literatures.

The third force, originating from the thermally activated fluctuation of a membrane fluctuating near a solid substrate, was described by Helfrich (1978):

$$\begin{array}{r} P_{und}\left(d\right)=\frac{\pi^{2}}{128}\frac{{\left(k_{B}T\right)}^{2}}{\kappa d^{3}}, \end{array}$$

κ is the bending modulus of the membrane, which is in the order of 10 k_B_T, while the pre-factor confirmed later by Monte Carlo simulations (Bachmann et al., 2001).

Rossetti et al. (2015) showed that the equilibrium membrane-substrate distance determined by specular neutron and X-ray reflectivity experiments could be reproduced quantitatively from the point of zero force determined by the summation of hydration repulsion, van der Waals interaction, and undulation force (Figure 3D). Intriguingly, we found that the disjoining pressure is dominated by the interplay of attractive van der Waals force and hydration repulsion, while the undulation force had a smaller contribution due to its shallower slope. A simple calculation suggests that the membrane cannot be decoupled from the substrate even at 60°C. In contrast, the membrane can readily be decoupled from thick or highly hydrated polymer supports, which seems to explain the previously reported budding of supported membranes on dextran supports that are swollen by a factor of 100–150. More recently, Kowalik et al. showed that the characteristic decay length of phospholipid membrane stacks significantly depends on the ordering of lipid molecules by quantitatively comparing the experimental pressure-distance relationships obtained by specular neutron reflectivity data and the atomistic molecular dynamics simulation (Kowalik et al., 2017). This report clearly demonstrates that the hydration force is not only caused by the ordering of water but also by the structural order of surfaces. As the conventional linear and non-linear optical spectroscopic techniques, such as second harnonic generation, are not able to separate the signals of interfacial water from bulk water, the use of X-ray absorption and X-ray emission spectroscopy has been drawing increasing attentions (Fransson et al., 2016). Although the optimization of sample environments with current instruments still seem non-trivial, such techniques will help researcher understand the structure of hydrating water near biological interfaces. Moreover, owing to the development of new instruments at spallation sources (Hino et al., 2013; Nickels and Katsaras, 2015), quasi elastic neutron scattering and neutron spin echo seem powerful to discriminate different modes of dynamics to gain more details into the dynamics of hydrating water. Using quasi elastic neutron scattering, Yamada et al. have demonstrated that the number of tightly bound water and loosely bound water to one lipid molecule actually depends on the structural phase (Yamada et al., 2017). Thus, the quantitative understanding of structures and dynamics of polymers and hydrating water will enable the rational design of material that can bridge hard solids and soft biological matters.

## Electrostatics of Soft Matter in Physiological Environments, Ion Specificity

In the previous section, one important interaction was not discussed, which is the electrostatic interaction. This is not negligible, as interfacial interactions in biological systems include a number of charged molecules. The surface of adult animal cells is coated with sialic acids, and extracellular matrix consists of many negatively charged macromolecules, such as glycosaminoglycan. However, it should be noted that the classical framework of Derjaguin-Landau-Verwey-Overbeek (DLVO) theory is no longer valid to handle electrostatics of soft matter in water. For example, the charge density in charged polymers is neither uniform nor static, and the DLVO theory cannot deal with the ion bridges between charged monomers mediated by multivalent ions, which even causes the coli-globule transition of DNA (Takahashi et al., 1997).

**Figure 4:**
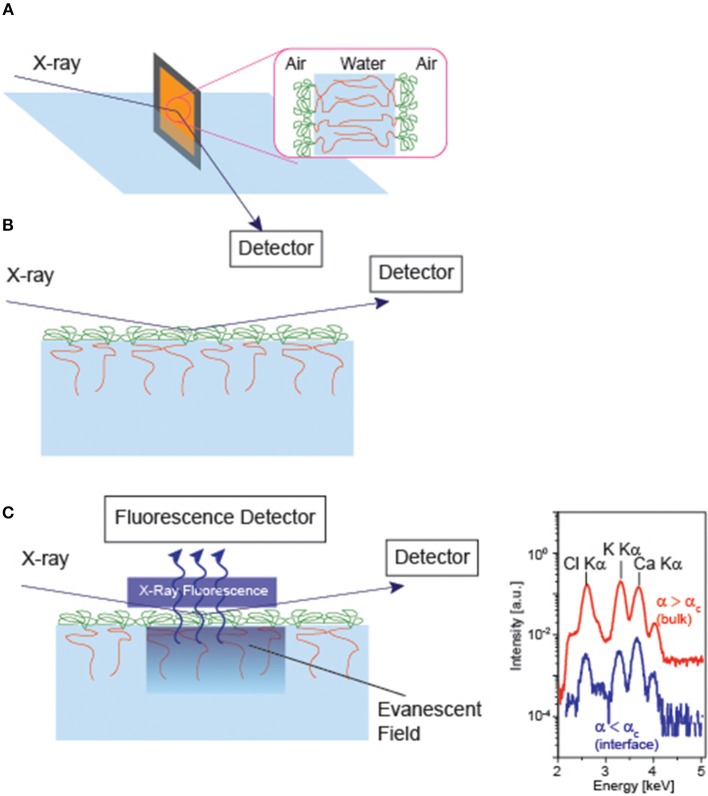
Specular X-ray reflectivity of **(A)** free-standing bilayer of diblock copolymer and **(B)** monolayer of diblock copolyme at the air/water interface. **(C)** Grazing incidence X-ray fluorescence of a monolyer at the air/water interface. The illumination of the sample at the incidence angles below and beyond the critical angle of total reflection (α_c_) enables one to tune the penetration depth of the illumination profile *I*_il_ (Schneck et al., 2010). From the X-ray fluorescence spectra collected at various incidence, the density profile of each element can be calculated with Å resolution. Note that the parallel measurement of specular reflectivity is necessary, as the illumination profile in nm-thick layers strongly depends on the electron densities of individual layers.

Experimentally, a number of groups have investigated how counter ions interact with charged polymers (Takahashi and Nagasawa, 1964; Hayashi et al., 2002; Biesalski et al., 2004; Ballauff and Borisov, 2006; Kobayashi et al., 2014). For example, (Guenoun et al., 1995) measured the thickness of free-standing diblock copolymers using specular X-ray reflectivity (Figure 4A), demonstrating that the thickness of polyelectrolyte layer *L* scales with the salt concentration *c* at high salt concentrations (*c* > 0.1 M), $L\;\propto \;c^{\frac{1}{3}}$. Ahrens et al. (1998) deposited the monolayer of di-block copolymer and measured the specular X-ray reflectivity at the air/water interface (Figure 4B). They found that the osmotically swollen polystyrene sulfonate brushes started shrinking only after the concentration of monovalent salts exceed 0.1 M. Moreover, the counter ion concentration amounts to 1 M, and about 90 % of counter ions are “bound” to the charged monomers, far beyond of Manning condensation (Manning, 1969).

Since the semantic report of Hofmeister in 1988 (Hofmeister, 1888) showing the order of various salts according to the capability to precipitate proteins from aqueous solutions (Jungwirth and Cremer, 2014), a number of studies have shown that the interfacial interactions in biological systems are ion specific. For example, the outer surface of Gram-negative bacteria is coated with negatively charged saccharide head groups of lipopolysaccharides (LPSs), and it has been known that bacteria increase become more resistant against cationic antimicrobial peptides in the presence of divalent cations (Ca^2+^, Mg^2+^) (Brock, 1958).

Despite the fact that X-ray and neutron reflectivity could provide with a rich variety of information about the electron and scattering length density profiles, these techniques cannot discriminate density profiles of monovalent and divalent ions in the vicinity of material/water interface. The application of grazing incidence X-ray fluorescence to the soft matter confined at the air/water interface (Figure 4 C) (Novikova et al., 2003) created a breakthrough, as it can discriminate the fluorescence emission from different core levels, such as K Kα and Ca Kα. By varying the incidence angle α_i_ near the angle of total reflection α_c_, one can modulate the penetration depth of the evanescence field Λ

$$\begin{array}{l} \Lambda \left(\alpha_{i}\right)=\frac{\lambda }{\sqrt{8}\pi }{\left[{\left({\left(\alpha_{i}^{2}-\alpha_{c}^{2}\right)}^{2}+4\beta \right)}^{\frac{1}{2}}-\left(\alpha_{i}^{2}-\alpha_{c}^{2}\right)\;\right]}^{\frac{1}{2}}, \end{array}$$

where β is the imaginary part of the refractive index, *n* = 1 − δ + *i*β. This means that the X-ray fluorescence recorded as a function of incidence angle *I*_fl_(α_i_) can be converted to the depth profile of the target element *c*(*z*),

$$\begin{array}{l} I_{fl}\left(\alpha_{i}\right)\propto \int^{\infty }I_{il}\left(\alpha_{i},z\right)c\left(z\right)\exp\left(-\frac{z}{L}\right)dz \end{array}$$

where *L* is the characteristic attenuation length of the element. This technique enables one to determine not only the density profiles of monovalent and divalent molecules (Schneck et al., 2010; Abuillan et al., 2013) but also the position of membrane bound proteins (Abuillan et al., 2012; Körner et al., 2013) within several Å resolution. It should be noted that the parallel measurement of the electron density profile by means of specular X-ray reflectivity is essential for such thin layers, because the illumination profile through the stratified interfaces *I*_il_(α_i_, *z*), which is nothing but the electromagnetic fields reflected and refracted at each interface.

In order to simulate interactions of diffusing ions with polymers in water and the resulting conformation changes of polymers, there are several requirements. Namely, the simulation volume must be large enough to deal with polymer layers with thickness of 10–100 nm, and must sample conformational changes occurring on time scales over 10^−3^ s. Netz wrote a minimum model for the charged interfaces and colloids (Netz, 1999), which enables one to carry out coarse-grained Monte Carlo simulation instead of atomistic models (Pink et al., 2003). In the case of bacteria surface models based on LPS monolayer, both GIXF data and Monte Carlo simulation suggest the replacement of K^+^ by Ca^2+^. As the degrees of dissociation of carboxyl and phosphate groups near the surface significantly depends on the electric potential at the surface (Coughlin et al., 1985), the combination of GIXF, coarse-grained simulations, and X-ray absorption/emmission spectroscopy (Fransson et al., 2016) seems promising for the precise determination of the ion density profiles near soft interfaces in aquatic environments.

## Toward Rational Design of New Materials: Bioinertness

One of the most intensively studied aspects of materials in water is the “bioinertness,” also called as “antifouling capability,” which is the capability of materials to avoid the non-specific protein adsorption and cell adhesion. This has a significant importance in a versatile of applications, ranging from the coating of medical devices such as contact lenses and implants to the protection of ships against the marine biofilms. The exact identification of proteins interacting with the material surface using the proteomic profiling becomes more important for biomedical applications because the body fluid, such as serum, contains various proteins (Hirohara et al., 2019). In recent several decades, a number of protein/cell-repelling materials have been developed, including poly- and oligoethylene glycols (Prime and Whitesides, 1993; Otsuka et al., 2012) and bioinspired dopamine-derivatives (Dalsin et al., 2005; Lee et al., 2007). More recently, zwitterionic materials, such as polymers with zwitterionic side chains, draw increasing attentions due to their excellent antifouling capability (Iwata et al., 2004; Zhang et al., 2006; Schlenoff, 2014; Shao and Jiang, 2015).

Yet the physical origins of bioinertness are still under debate. The electrostatic interactions seem to matter, but do not seem to play dominant roles because negatively charged surfaces could cause fouling. As described the previous section, it is still challanging to deal with ion specific interactions of charged polymers. For example, Wang et al. reported the anion-specific changes in conformation and protein repellency for poly-sulfobetaine follow Hofmeister series (Wang et al., 2013), while Higaki et al. suggested that the dependence of effective interaction potentials on cations cannot be explained by this framework (Higaki et al., 2017). It also seems plausible that the conformational fluctuation of molecules entropically repells proteins and cells from the surface. However, many studies have shown that monolayers based on short oligo ethylene glycols possess excellent bioinertness (Harder et al., 1998). Although vibrational spectroscopy data suggest that the structures of water in contact with such bioinert surfaces are different from bulk water (Morita et al., 2007), the ensemble information from the spectroscopy cannot resolve the thickness and density of interfacial water have not bee resolved. Recent interfacial force measurements have shown several interesting findings. Hayashi et al. reported the presence of several nm-thick, repulsive layers near the bioinert oligoethylene glycol surfaces (Hayashi et al., 2012), and Shi et al. demonstrated there is a long-range attraction between water and zwitterionic polymer brushes in oil (Shi et al., 2016). Further studies on the structures and dynamics of water in the vicinity of polymer/water interface potentially enable to overcome the commonly taken trial-and-error approaches toward the rational design, or even the prediction of new bioinert materials.

“How interfacial water modulates biological and soft matters” is not yet an answered question. The quantification of structure-function relationships using defined model systems and multiscale simulations would help us rationally design the new materials, which do not only help us understand the basic principles regulating functions of proteins and biopolymers in biology but also create new aquatic materials that can even overcome the function of biomolecules by fine-tuning of interfacial interactions with water.

## Author Contributions

MT designed and wrote this review article.

### Conflict of Interest

The author declares that the research was conducted in the absence of any commercial or financial relationships that could be construed as a potential conflict of interest.
